# Current Status and Unmet Needs of EUS Education in Japan: A Nationwide Survey Focusing on Screening EUS, EUS‐Guided Sampling, and Interventional EUS

**DOI:** 10.1002/deo2.70430

**Published:** 2026-09-10

**Authors:** Susumu Hijioka, Ichiro Yasuda, Atsushi Irisawa, Akio Katanuma, Shomei Ryozawa, Masayuki Kitano, Hiroyuki Isayama, Kazuo Hara, Yousuke Nakai, Takuji Iwashita, Daiki Yamashige, Takuji Okusaka, Takao Itoi

**Affiliations:** ^1^ Department of Hepatobiliary and Pancreatic Oncology National Cancer Center Hospital Tokyo Japan; ^2^ Third Department of Internal Medicine University of Toyama Toyama Japan; ^3^ Department of Gastroenterology School of Medicine Dokkyo Medical University Mibu Tochigi Japan; ^4^ Department of Gastroenterology and Hepatology School of Medicine Sapporo Medical University Sapporo Hokkaido Japan; ^5^ Department of Gastroenterology Saitama Medical University International Medical Center Saitama Japan; ^6^ Second Department of Internal Medicine Wakayama Medical University Wakayama Japan; ^7^ Department of Gastroenterology, Graduate School of Medicine Juntendo University Tokyo Japan; ^8^ Department of Gastroenterology Aichi Cancer Center Hospital Nagoya Aichi Japan; ^9^ Department of Internal Medicine Institute of Gastroenterology Tokyo Women's Medical University Tokyo Japan; ^10^ Department of Gastroenterology Shiga University of Medical Science Shiga Japan; ^11^ Department of Gastroenterology and Hepatology Tokyo Medical University Tokyo Japan

**Keywords:** competency‐based training, endoscopic ultrasound, interventional endoscopic ultrasound, medical education, simulation‐based training

## Abstract

**Objectives:**

Endoscopic ultrasound (EUS) plays a central role in the management of pancreaticobiliary diseases. However, EUS training in Japan remains highly dependent on the experience and educational capacity of individual institutions and supervisors, and the nationwide status of EUS education has not been comprehensively evaluated. This study investigated the current status and unmet educational needs of EUS training in Japan through a nationwide survey.

**Methods:**

An anonymous nationwide questionnaire was distributed to 120 institutions performing EUS across Japan through the investigators’ professional network. Institutions routinely performing diagnostic and/or therapeutic EUS were invited to participate in the survey. Survey items included institutional characteristics and educational practices related to screening EUS, EUS‐guided sampling, and interventional EUS (I‐EUS). Data regarding annual procedure volumes, criteria for trainee initiation, training systems, and educational challenges were collected.

**Results:**

In total, 114 institutions participated. Median annual procedure volumes were 510 for screening EUS, 135 for EUS‐guided sampling, and 25 for I‐EUS. The number of supervising physicians increased with procedural complexity. Most institutions reported that I‐EUS was performed by physicians with >10 years of clinical experience. Criteria for trainee initiation varied substantially among institutions, and no standardized educational pathway was identified. Major challenges included limited case volume, concerns regarding adverse events, and a lack of simulation‐based training resources. Only three institutions reported permanent access to dedicated phantom models.

**Conclusions:**

EUS education in Japan remains largely apprenticeship‐based, with considerable interinstitutional variability. Standardized curricula, objective competency assessment, and simulation‐based training systems are needed to optimize future EUS education.

## Graphical abstract

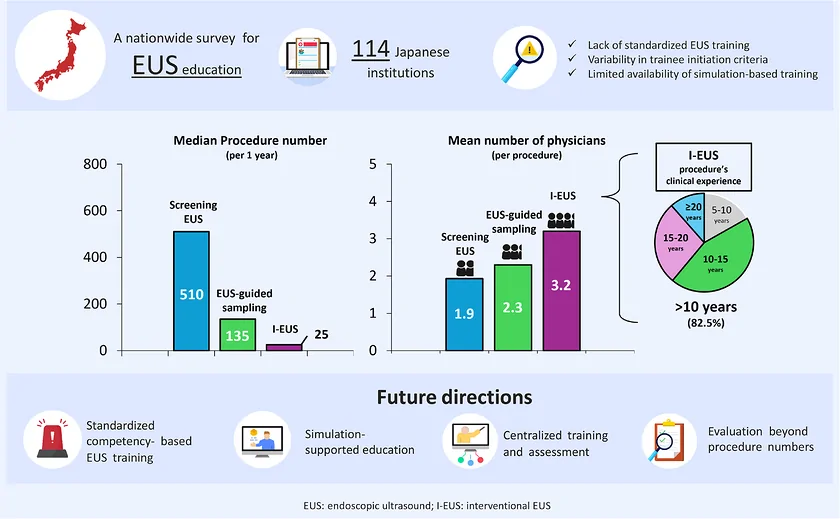

## 1 Introduction

Endoscopic ultrasound (EUS) has become an essential modality for the diagnosis and management of gastrointestinal diseases, particularly pancreaticobiliary disorders. In recent years, EUS‐related procedures have expanded beyond diagnostic imaging to include EUS‐guided sampling and interventional EUS (I‐EUS), broadening their role from diagnosis to therapeutic intervention [1]. However, EUS is a technically demanding procedure that requires advanced anatomical knowledge and sophisticated endoscopic manipulation skills, and proficiency achievement generally requires substantial training and experience.

Traditionally, EUS education has relied on an apprenticeship‐based model, in which trainees acquire procedural skills through observation and supervised practice under expert endoscopists. Although this approach facilitates the transfer of tacit knowledge and clinical judgment, educational content and assessment standards often depend on individual institutions and trainers, limiting reproducibility, educational efficiency, and standardization. In contrast, surgical fields, particularly robotic surgery, have increasingly adopted structured training programs incorporating simulators, objective assessment tools, and competency‐based frameworks. However, standardized educational models and objective competency assessment systems for EUS remain insufficiently developed [2, 3].

I‐EUS, in particular, requires advanced device‐handling skills and the ability to manage potentially severe adverse events. However, limited procedural volume at individual institutions may restrict trainee exposure and opportunities for skill acquisition. In addition, criteria for trainee initiation and competency achievement vary considerably among institutions, and no clear consensus has been established. As EUS‐related procedures continue to increase in complexity, optimization of educational systems has become increasingly important.

Previous studies on EUS education have largely been limited to small single‐center or regional investigations, and recent systematic reviews and surveys have highlighted substantial heterogeneity in training structures and competency definitions across institutions and countries [4, 5, 6]. To our knowledge, comprehensive nationwide evaluations of EUS education, particularly those encompassing screening EUS, EUS‐guided sampling, and I‐EUS, remain scarce in Asian countries. Therefore, this nationwide survey aimed to clarify the current status of EUS education in Japan and identify unmet needs and future directions for the standardization and optimization of EUS training systems.

## 2 Methods

### 2.1 Study Design and Survey Administration

This multicenter cross‐sectional survey was conducted to evaluate the current status of EUS education in Japan. An anonymous nationwide questionnaire was distributed to 120 institutions performing EUS to assess educational systems related to screening EUS, EUS‐guided sampling, and I‐EUS, including trainee initiation criteria, supervisory structures, staffing, and educational challenges. The survey was administered using a web‐based platform, and responses were collected between May and June 2025.

Participating institutions included university hospitals, cancer centers, and regional core hospitals with diverse clinical backgrounds. One response per institution was accepted, and each questionnaire was completed by a representative physician involved in EUS education. A total of 114 institutions participated in the survey. Duplicate responses were excluded from the analysis. Terminology for EUS‐related procedures was used according to the classification of interventional endoscopic ultrasonography/endosonography proposed by the Japan Gastroenterological Endoscopy Society [7]. Screening EUS was defined as diagnostic EUS performed primarily for lesion detection and morphological assessment. EUS‐guided sampling was defined as EUS‐guided tissue acquisition using a needle, including EUS‐guided fine‐needle aspiration and fine‐needle biopsy. I‐EUS included therapeutic EUS procedures excluding EUS‐guided sampling. In this survey, trainees were defined as physicians undergoing EUS training at each institution, pancreaticobiliary specialists as physicians primarily engaged in pancreaticobiliary endoscopy or the management of pancreaticobiliary diseases, and supervising physicians as physicians responsible for performing EUS procedures and supervising EUS training. These categories were classified according to the judgment of the respondent at each participating institution.

Although participating institutions were known to the study investigators, survey responses were collectively analyzed, and no institution‐specific data are presented in this report.

This survey evaluated institutional educational systems and did not collect any patient information, clinical data, or identifiable personal information. Therefore, according to the Japanese Ethical Guidelines for Medical and Health Research Involving Human Subjects, institutional review board approval was not required.

### 2.2 Questionnaire Development

The questionnaire was developed through discussions among the authors, all with extensive clinical and educational experience in pancreaticobiliary endoscopy, including diagnostic EUS, EUS‐guided sampling, and I‐EUS. Responses were collected using a web‐based survey platform, and only one response per institution was accepted. Multiple responses to each item were not allowed. Free‐text fields were included for selected items. The questionnaire comprised the following four domains:

1. Institutional characteristics
  Type of institution (university hospital, regional core hospital, or cancer center), number of hospital beds, number of full‐time gastroenterologists, number of pancreaticobiliary specialists, and number of supervising physicians.
2. Screening EUS
  Annual procedure volume, scope types (linear‐array and/or radial type), trainee initiation criteria, educational methods, procedural staffing, competency goals, and educational challenges.
3. EUS‐guided sampling
  Annual procedure volume, initiation criteria, procedural staffing, criteria for switching procedures to supervising physicians, and educational challenges.
4. I‐EUS
  Annual procedure volume, initiation criteria, postgraduate years of operators, required EUS‐guided sampling experience before I‐EUS training, number of cases considered necessary to achieve “expert hand” status, supervisory systems, and educational challenges.

Several items included free‐text response fields to obtain detailed information regarding educational strategies, practical approaches, and institutional challenges. The full questionnaire has been added as Supporting Information.

### 2.3 Outcomes

The primary outcomes were current educational systems and educational challenges associated with each EUS‐related procedure, including screening EUS, EUS‐guided sampling, and I‐EUS. Analyzed items were as follows:

- Annual procedure volumes for each EUS‐related procedure
- Criteria for trainee initiation
- Procedural staffing
- Number of cases considered necessary to achieve “expert hand” status
- Educational challenges and strategies (free‐text responses)

The number of physicians involved in each procedure refers to the number of physicians directly involved in that procedure; nurses and other staff were excluded. The free‐text responses were independently reviewed by two authors (S.H. and D.Y.), and similar opinions and comments were categorized through discussion until consensus was reached to identify common educational challenges and institutional strategies.

### 2.4 Statistical Analysis

Continuous variables are expressed as medians with interquartile ranges (IQRs) because of the potential for non‐normal distributions. Mean values and 95% confidence intervals (95% CIs) were also calculated as reference values. Categorical variables are presented as numbers and percentages, using all 114 participating institutions as the denominator. Missing data were not imputed and shown as “No response” when applicable. Continuous variables were analyzed using available numerical responses.

Free‐text responses were systematically reviewed, and similar responses were grouped into categories for descriptive analysis. Statistical analyses were primarily descriptive. Because the purpose of this study was exploratory evaluation of the current status of EUS education, no comparative analyses or hypothesis testing were performed. Data were analyzed using IBM SPSS Statistics version 31.0 (IBM Corp., Armonk, New York, USA).

## 3 Results

### 3.1 Participating Facilities and Baseline Characteristics

**FIGURE 1:**
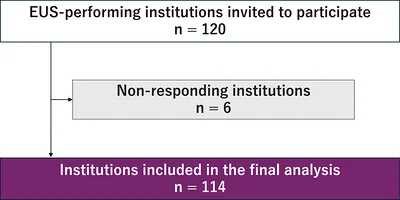
Flowchart showing the participation of facilities in the nationwide questionnaire survey. Endoscopic ultrasound (EUS)

Among the 120 institutions invited to participate, 114 responded and were included in the final analysis (response rate, 95.0%). Six institutions did not respond and were excluded (Figure 1). Participating institutions included 63 university hospitals (55.3%), 40 regional core hospitals (35.1%), and 11 cancer centers (9.6%). These institutions represented diverse clinical settings and broadly reflected real‐world EUS educational practices in Japan (Table 1).

**TABLE 1:** Institutional characteristics.

Institution type	Number of institutions (*n* = 114)
University hospitals	63 (55.3%)
Regional core hospitals	40 (35.1%)
Cancer centers	11 (9.6%)

### 3.2 Education for Screening EUS

**FIGURE 2:**
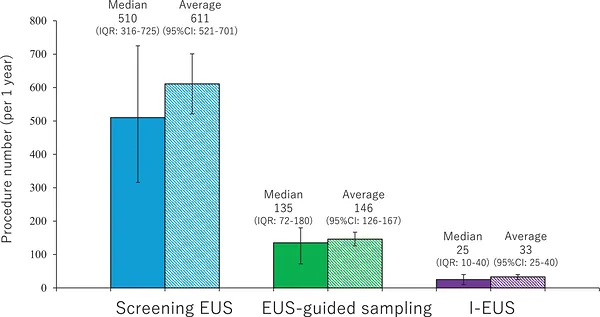
Annual procedural volume of screening EUS, EUS‐guided sampling, and I‐EUS among participating institutions. The median annual procedural volumes were 510 (IQR, 316–725) for screening EUS, 135 (IQR, 72–180) for EUS‐guided sampling, and 25 (IQR, 10–40) for I‐EUS. Mean annual volumes were 611 (95% CI, 521–701), 146 (95% CI, 126–167), and 33 (95% CI, 25–40), respectively. CI, confidence interval; EUS, endoscopic ultrasound; I‐EUS, interventional EUS; IQR, interquartile range.

**FIGURE 3:**
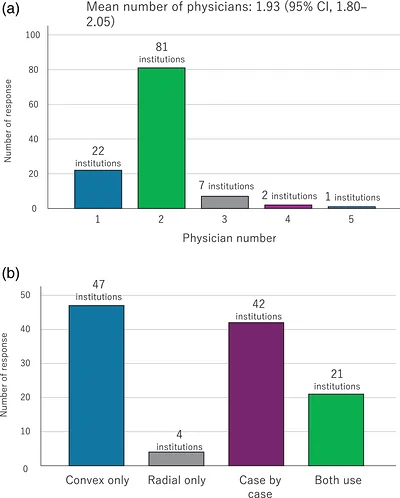
Number of physicians involved during screening EUS procedures (a) and types of echoendoscopes used for screening EUS (b). (a) The mean number of physicians involved in screening EUS procedures was 1.93 (95% CI, 1.80–2.05); this indicates that most procedures were performed by one or two physicians. One institution did not respond. (b) Among the participating institutions, 47 (41.2%) exclusively used linear‐array echoendoscopes, 42 (36.8%) selected linear‐array or radial echoendoscopes according to the clinical situation, 21 (18.4%) routinely used both types, and 4 (3.5%) exclusively used radial echoendoscopes. “Both” indicates institutions that routinely used both linear‐array and radial echoendoscopes, whereas “case by case” indicates institutions that selected echoendoscopes according to the clinical situation. CI, confidence interval; EUS, endoscopic ultrasound.

The median annual number of screening EUS procedures per institution was 510 (IQR, 316–725), and the mean number was 611 (95% CI, 521–701) (Figure 2). The mean number of physicians involved in screening EUS procedures was 1.93 (95% CI, 1.80–2.05) (Figure 3a). Regarding scope selection, 47 institutions (41.2%) used only linear‐array echoendoscopes, 42 (36.8%) selected linear‐array or radial scopes according to the clinical situation, 21 (18.4%) routinely used both scope types, and 4 (3.5%) used only radial scopes (Figure 3b).

Few institutions had clearly defined case‐volume thresholds for initiating training for EUS screening. Instead, many institutions considered postgraduate year, typically Years 4–5, or independent competency in screening upper and lower gastrointestinal endoscopy as prerequisites for trainee initiation (Table 2). Only three institutions (2.6%) reported permanent availability of dedicated phantom models for simulation‐based training.

**TABLE 2:** Criteria for initiating screening endoscopic ultrasound (EUS) training.

Criteria	Number of institutions (*n* = 114)
Postgraduate year criteria (typically after achieving independent competency in upper and lower gastrointestinal endoscopy, around postgraduate Years 4–5)	40 (35.1%)
After sufficient observation and assistant experience in EUS	20 (17.5%)
Subjective judgment by supervising physicians	11 (9.6%)
Others (motivation regardless of years of experience, etc.)	29 (25.4%)
No response	14 (12.3%)

Educational challenges related to screening EUS varied among institutions (Table 3). Commonly reported issues included difficulty securing adequate training time because the procedure time is longer for screening EUS than for screening upper or lower gastrointestinal endoscopy, as well as inconsistency in instructional approaches among supervisors.

**TABLE 3:** Educational challenges in screening endoscopic ultrasound (EUS).

Educational challenge	Number of institutions (*n* = 114)
Insufficient training time because screening EUS requires longer procedure times than screening upper or lower gastrointestinal endoscopy	30 (26.3%)
Inconsistency in teaching methods among supervisors	25 (21.9%)
Lack of suitable phantom models	6 (5.3%)
Difficulty reviewing examination techniques	3 (2.6%)
Others (shortage of supervisors, difficulty teaching, etc.)	42 (36.8%)
No response	8 (7.0%)

### 3.3 Education for EUS‐Guided Sampling

**FIGURE 4:**
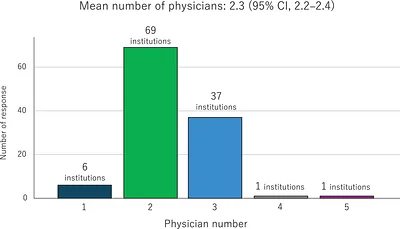
Number of physicians involved during EUS‐guided sampling procedures. The mean number of physicians involved in EUS‐guided sampling procedures was 2.3 (95% CI, 2.2–2.4); this reflects a more intensive supervisory structure than that used for screening EUS. CI, confidence interval; EUS, endoscopic ultrasound.

The median annual number of EUS‐guided sampling procedures was 135 (IQR, 72–180), and the mean number was 146 (95% CI, 126–167) (Figure 2). The mean number of physicians involved during EUS‐guided sampling procedures was 2.3 (95% CI, 2.2–2.4), indicating that EUS‐guided sampling was generally performed under a more intensive supervisory system than was screening EUS (Figure 4).

Regarding trainee initiation criteria for EUS‐guided sampling, 46 institutions (40.4%) considered independent performance of screening EUS a prerequisite. However, standardized initiation criteria were not identified, and substantial interinstitutional variability was observed, including reliance on subjective assessment by supervising physicians (Table 4).

**TABLE 4:** Criteria for initiating endoscopic ultrasound (EUS)‐guided sampling training.

Criteria	Number of institutions (*n* = 114)
Independent performance of screening EUS	46 (40.4%)
Subjective judgment by supervising physicians	23 (20.2%)
Ability to consistently visualize the target lesion without completing the entire screening examination	13 (11.4%)
After some experience with screening EUS	5 (4.4%)
Others (screening EUS from the stomach only, motivation‐dependent, postgraduate year ≥3, etc.)	26 (22.8%)
No response	1 (0.9%)

Major educational challenges associated with EUS‐guided sampling included insufficient case volume, limited opportunities for procedural experience and hands‐on training, difficulty teaching procedural techniques, and lack of suitable phantom models for training (Table 5).

**TABLE 5:** Educational challenges in endoscopic ultrasound (EUS)‐guided sampling.

Educational challenge	Number of institutions (*n* = 114)
Insufficient case volume and procedural experience	44 (38.6%)
Difficulty teaching procedural techniques	12 (10.5%)
Lack of suitable phantom models	3 (2.6%)
Limited opportunities to learn device‐related knowledge	2 (1.8%)
Lack of established educational systems	6 (5.3%)
Others (difficulty supervising while performing procedures, etc.)	23 (20.2%)
No response	24 (21.1%)

### 3.4 Education for I‐EUS

**FIGURE 5:**
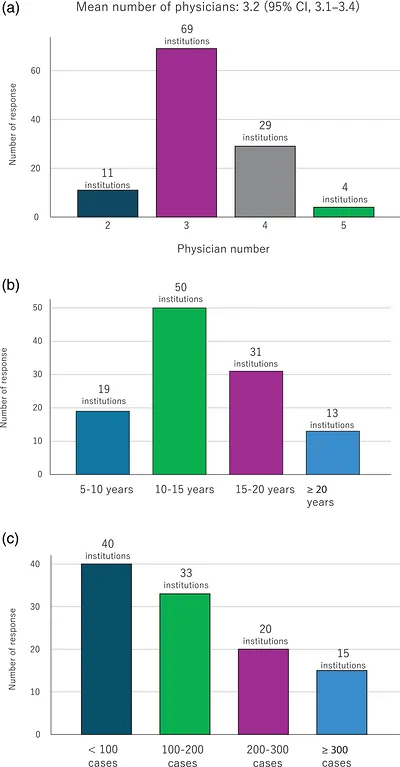
Number of physicians involved during I‐EUS procedures (a), postgraduate years of physicians performing I‐EUS (b), and previous EUS‐guided sampling experience among I‐EUS operators. (a) The mean number of physicians involved in I‐EUS procedures was 3.2 (95% CI, 3.1–3.4); this demonstrates the implementation of a more manpower‐intensive supervisory system for advanced therapeutic procedures. One institution did not respond. (b) Physicians with 10–15 years of postgraduate experience represented the largest group, followed by those with 15–20 years of experience. This indicates that I‐EUS was predominantly performed by highly experienced endoscopists. One institution did not respond. (c) Regarding previous EUS‐guided sampling experience among I‐EUS operators, the largest proportion of institutions reported <100 EUS‐guided sampling cases, followed by 100–200 cases. Six institutions did not respond. CI, confidence interval; EUS, endoscopic ultrasound; I‐EUS, interventional EUS.

The median annual number of I‐EUS procedures, excluding EUS‐guided sampling, was 25 (IQR, 10–40), and the mean number was 33 (95% CI, 25–40) (Figure 1). The mean number of physicians involved during I‐EUS procedures was 3.2 (95% CI, 3.1–3.4), demonstrating that I‐EUS was performed under a more cautious and manpower‐intensive supervisory system than was screening EUS and EUS‐guided sampling (Figure 5a).

Regarding operator experience, physicians with 10–15 years of postgraduate experience represented the largest group (50 institutions, 43.9%), followed by those with 15–20 years of experience (31 institutions, 27.2%). These findings indicate that I‐EUS was predominantly performed by physicians with >10 years of clinical experience (94 institutions, 82.5%) (Figure 5b).

For initiation of I‐EUS training, the most common prerequisite was independent performance of EUS‐guided sampling (42 institutions, 36.8%), followed by the ability to assist with I‐EUS procedures in addition to independently performing EUS‐guided sampling (13 institutions, 11.4%). However, no clear consensus regarding trainee initiation criteria was identified (Table 6). Regarding previous EUS‐guided sampling experience among I‐EUS operators, the largest proportion of institutions reported <100 EUS‐guided sampling cases (40 institutions, 35.1%), followed by 100–200 cases (33 institutions, 28.9%) (Figure 5c).

**TABLE 6:** Criteria for initiating interventional EUS (I‐EUS) training.

Criteria	Number of institutions (*n* = 114)
Independent performance of EUS‐guided sampling	42 (36.8%)
Ability to assist with I‐EUS in addition to independently performing EUS‐guided sampling	13 (11.4%)
Ability to perform all pancreaticobiliary procedures except I‐EUS	11 (9.6%)
Experience with ≥50 EUS‐guided sampling cases	11 (9.6%)
Experience with ≥100 EUS‐guided sampling cases	6 (5.3%)
No educational system; procedures performed only by supervisors	6 (5.3%)
Experience with 10–30 EUS‐guided sampling cases	4 (3.5%)
Others (criteria unclear, etc.)	18 (15.8%)
No response	3 (2.6%)

Major educational challenges associated with I‐EUS included reduced case exposure at individual institutions due to case distribution across centers, reluctance to allow trainee participation because of the risk of severe adverse events, and difficulty adapting to rapidly evolving devices and procedural techniques (Table 7).

**TABLE 7:** Educational challenges in interventional EUS (I‐EUS).

Educational challenge	Number of institutions (*n* = 114)
Limited case exposure at individual institutions due to case dispersion	47 (41.2%)
Hesitation regarding trainee involvement because of the potential for severe adverse events	9 (7.9%)
Difficulty adapting to rapidly evolving devices	6 (5.3%)
Lack of simulation training using phantom models	3 (2.6%)
Difficulty predicting procedural difficulty in advance	2 (1.8%)
Lack of supervisory systems	1 (0.9%)
Other	35 (30.7%)
No response	11 (9.6%)

## 4 Discussion

This nationwide survey of EUS‐performing institutions in Japan is, to our knowledge, the first large‐scale study to comprehensively evaluate educational systems across EUS procedures with different levels of complexity, including screening EUS, EUS‐guided sampling, and I‐EUS. Recent systematic reviews and position statements have summarized available EUS training curricula and highlighted considerable heterogeneity in training structures and competency definitions across regions; however, most data derive from single‐center or non‐nationwide studies and focus primarily on diagnostic EUS or EUS‐guided sampling rather than the full spectrum of EUS‐related procedures [2, 3, 4, 6]. Moreover, to our knowledge, this is the first nationwide comprehensive survey from Asia to evaluate educational systems spanning screening EUS, EUS‐guided sampling, and I‐EUS.

Substantial interinstitutional variability was observed in trainee initiation criteria, supervisory structures, and competency goals for each EUS‐related procedure. These findings likely reflect the long‐standing dependence of EUS education in Japan on apprenticeship‐based training shaped by the experience and educational philosophy of individual institutions and supervisors. Similar variability in learning curves and competence achievement among advanced endoscopy trainees has been reported in multicenter studies of EUS and endoscopic retrograde cholangiopancreatography training, underscoring the limitations of unstructured apprenticeship models and the need for more standardized, competence‐based education [5, 8].

Another important observation was the stepwise change in educational systems according to procedural complexity. As procedures progressed from screening EUS to EUS‐guided sampling and, subsequently, I‐EUS, the number of physicians involved during procedures increased, indicating implementation of progressively more cautious supervisory systems. However, in reality, six facilities reported that EUS‐guided sampling was performed by a single physician. This does not mean that the procedure was performed entirely by the physician alone; although one physician was directly involved as the operator, the procedure was actually carried out with the support of nurses and other medical staff. Given the existence of such arrangements, EUS training must consider not only physicians but also the support system involving a multidisciplinary team. I‐EUS was predominantly performed by physicians with ≥10 years of postgraduate clinical experience. In addition, limited institutional case volumes and concerns regarding severe adverse events appeared to restrict educational opportunities for younger physicians. Although independent performance of EUS‐guided sampling was frequently considered a prerequisite for I‐EUS training, the criteria used for trainee evaluation remained largely subjective and varied substantially among institutions, highlighting the lack of a clear national consensus. In line with previous reports emphasizing the technical complexity and potential for serious adverse events with I‐EUS, our findings highlight that I‐EUS remains largely concentrated among highly experienced operators, and that low case volumes at individual centers may further constrain opportunities for structured hands‐on training [1, 9, 10].

One of the most notable findings was the limited implementation of objective competency assessment systems and simulation‐based education in EUS training. Only 3 of 114 institutions reported permanent availability of dedicated phantom models, and simulator‐based off‐the‐job training had not become widely established. This is consistent with recent systematic reviews indicating that, although various EUS competency assessment tools and training models have been proposed, evidence regarding validity remains limited and routine integration into training programs remains uncommon [4, 9, 10, 11]. Nevertheless, free‐text responses identified several attempts to structure and visualize EUS education, including video review, procedural annotation, self‐assessment tools, and debriefing systems. In recent years, educational digital transformation (educational DX) has rapidly progressed across medical education, and in the field of EUS, structured educational models incorporating simulators, video‐based educational materials, and online learning platforms may therefore assume an increasingly important role in future training systems.

Centralization of educational systems may also represent a practical strategy for addressing limited case exposure in I‐EUS training. Rather than requiring all institutions to independently provide advanced I‐EUS education, establishment of dedicated training centers with sufficient procedural volume and experienced supervisors may improve educational quality and training efficiency.

However, this study has several limitations. First, participating institutions were recruited through the investigators’ professional network. Therefore, institutions with greater experience or interest in EUS practice and education may have been overrepresented, and selection bias cannot be excluded. Accordingly, the findings should be interpreted with caution before generalization to EUS educational practices across Japan.

Second, this study did not identify the total number of facilities performing EUS in Japan, and the participating facilities may not fully represent all such facilities nationwide. Furthermore, because the survey targeted facilities known to perform EUS‐related procedures, facilities with a high number of cases or a strong interest in EUS education were probably more likely to be included; therefore, selection bias and nonresponse bias cannot be excluded. On the other hand, this survey included 114 facilities with diverse geographic distributions and institutional backgrounds, and the survey nevertheless provides important empirical data for understanding the current status and challenges of EUS education in Japan. Third, this study did not directly evaluate educational quality or clinical outcomes associated with specific training systems. Future studies investigating associations between educational interventions and clinical performance outcomes are therefore necessary.

Although EUS education in Japan has developed primarily through institution‐based experiential learning, standardization of educational systems and competency assessment appears to remain insufficient. On the basis of our survey findings, a stepwise educational roadmap for EUS training in Japan is presented as a potential framework for future educational development. It incorporates structured supervision, simulation‐based training, and objective competency assessment to facilitate progressive skill acquisition for screening EUS, EUS‐guided tissue acquisition, and I‐EUS (Figure S1). These findings may provide a foundation for the future development of structured educational models. Standardization of training systems, integration of digital technologies, and establishment of specialized educational centers may further improve the quality and efficiency of EUS education.

## Author Contributions

**Susumu Hijioka**: conceptualization, study design, survey development, data analysis, manuscript drafting. **Ichiro Yasuda**: survey development, critical revision of the manuscript, and final approval. **Atsushi Irisawa**: survey development, critical revision of the manuscript, and final approval. **Akio Katanuma**: survey development, critical revision of the manuscript, and final approval. **Shomei Ryozawa**: survey development, critical revision of the manuscript, and final approval. **Masayuki Kitano**: survey development, critical revision of the manuscript, and final approval. **Hiroyuki Isayama**: survey development, critical revision of the manuscript, and final approval. **Kazuo Hara**: survey development, critical revision of the manuscript, and final approval. **Yousuke Nakai**: survey development, critical revision of the manuscript, and final approval. **Takuji Iwashita**: survey development, critical revision of the manuscript, and final approval. **Daiki Yamashige**: data collection, data analysis, manuscript revision. **Takuji Okusaka**: study supervision, manuscript revision, and final approval. **Takao Itoi**: conceptualization, study supervision, critical revision of the manuscript, and final approval.

## Funding

The authors have nothing to report.

## Ethics Statement

Approval of the research protocol: Institutional review board approval was not required because this anonymous survey did not collect patient information, clinical data, or identifiable personal information.

## Consent

The authors have nothing to report.

## Conflicts of Interest

The authors declare no conflicts of interest.

## Supporting information




**Supplementary Methods**: Full Questionnaire

**Supplementary Figure S1**: Proposed Competency‐based Educational Roadmap for EUS Training in Japan. EUS, Endoscopic Ultrasound.

## Data Availability

The data that support the findings of this study are available on request from the corresponding author. The data are not publicly available due to privacy or ethical restrictions.
