# Supplementary material for: Current Status and Unmet Needs of EUS Education in Japan: A Nationwide Survey Focusing on Screening EUS, EUS‐Guided Sampling, and Interventional EUS
Source: DEN Open. 2026 Sep 10;7(1):e70430. doi: 10.1002/deo2.70430 (PMC13562805; doi:10.1002/deo2.70430)
Supplement: Supplementary file 1 [file DEO2-7-e70430-s002.docx]

**Supplementary Material**

**Full questionnaire**

**Institutional background**

1. **Please enter the name of your institution.**
2. **Please select the category of your institution.**
   - **University hospital**
   - **Cancer center**
   - **Regional core hospital**
   - **Other: free-text response**
3. **Please enter the total number of hospital beds at your institution.**
4. **Please enter the number of full-time physicians in the Department of Gastroenterology.**
5. **Please enter the number of board-certified trainers of the Japan Gastroenterological Endoscopy Society at your institution.**
6. **Please enter the number of full-time physicians specializing in pancreaticobiliary diseases.**
7. **Please enter the number of physicians who supervise pancreaticobiliary endoscopy training.**

**Items regarding screening EUS**

1. **Please indicate the approximate annual number of EUS procedures performed at your institution.**
2. **Please indicate the type of echoendoscope used for screening EUS.**
   - **Radial echoendoscope**
   - **Convex echoendoscope**
   - **Both scopes routinely used**
   - **Case by Case**
   - **Other: free-text response**
3. **Please describe any approximate criteria used to determine when trainees can begin performing screening EUS. (Examples: years of clinical experience, judgment of each supervisor, number of observed or assisted procedures.)**
4. **Does your institution have routine access to an EUS phantom model?**
5. **Before on-the-job training, does your institution routinely provide ex vivo training using a phantom model or simulator?**
6. **How many physicians are usually involved when performing screening EUS?**
7. **When teaching beginners, does your institution separately teach gastric scanning and duodenal scanning?**
8. **Does your institution have any indicators or objective targets for determining competency in screening EUS?
   Free-text response.**
9. **Are there any educational challenges in teaching screening EUS at your institution?
   Free-text response.**

**Items regarding EUS-guided sampling**

1. **Please indicate the approximate annual number of EUS-FNA/FNB procedures performed at your institution.**
2. **Please describe any approximate criteria used to determine when trainees can begin performing EUS-FNA/FNB.
   (Examples: number of screening EUS procedures performed, judgment of each supervisor, number of observed or assisted EUS-FNA/FNB procedures.)**
3. **How many physicians are usually involved when performing EUS-FNA/FNB?**
4. **Under what circumstances does the supervisor take over the procedure from a trainee?
   Free-text response.**
5. **Are there any educational challenges in teaching EUS-FNA/FNB at your institution?
   Free-text response.**

**Items regarding interventional EUS**

**I-EUS, excluding EUS-guided sampling**

1. **Please indicate the approximate annual number of I-EUS procedures performed at your institution.**
2. **Please describe any approximate criteria used to determine when trainees can begin performing I-EUS.
   (Examples: number of EUS-FNA/FNB procedures performed, judgment of each supervisor, number of observed or assisted procedures.)**
3. **How many physicians are usually involved when performing I-EUS?**
4. **Approximately how many years of clinical experience do physicians usually have when they perform I-EUS at your institution?**
5. **At your institution, how many EUS-FNA/FNB procedures are generally required before physicians perform I-EUS?**
6. **Please indicate the number of I-EUS cases that your institution considers necessary to define an “expert hand.”**
7. **Under what circumstances does the supervisor take over the procedure from a trainee?
   Free-text response.**
8. **Are there any educational challenges in teaching I-EUS at your institution?
   Free-text response.**

**Final question**

1. **Please describe any particularly successful initiatives or approaches in EUS education at your institution.
   Free-text response.**
